# The First Data on the Complete Genome of a Tetrodotoxin-Producing Bacterium

**DOI:** 10.3390/toxins13060410

**Published:** 2021-06-09

**Authors:** Daria I. Melnikova, Reindert Nijland, Timur Yu. Magarlamov

**Affiliations:** 1 A.V. Zhirmunsky National Scientific Center of Marine Biology, Far Eastern Branch, Russian Academy of Sciences, 690041 Vladivostok, Russia; dmelnikova@imb.dvo.ru; 2 Marine Animal Ecology Group, Department of Animal Sciences, Wageningen University and Research, P.O. Box 338, 6700 AH Wageningen, The Netherlands; reindert.nijland@wur.nl

**Keywords:** tetrodotoxin (TTX), TTX-producing bacteria, genome

## Abstract

Tetrodotoxin (TTX)-producing bacteria have attracted great interest as a model system for study of the TTX biosynthetic route. Here, we report the complete genome of the TTX-producing bacterium *Bacillus* sp. 1839. The genome of the strain *Bacillus* sp. 1839, previously isolated from the TTX-bearing marine ribbon worm *Cephalothrix* cf. *simula*, was obtained using second generation Illumina and third generation nanopore sequencing technologies. Phylogenetic analysis has classified this strain as *Cytobacillus gottheilii*.

## 1. Introduction

Tetrodotoxin (TTX), broadly distributed in marine ecosystems, is one of the most studied neurotoxins of the 20th–21st centuries [1]. Its ability to selectively bind voltage-gated sodium channels resulted in the popularity of the toxin in medical, pharmaceutical, and scientific spheres [2]. Despite the wide distribution, the molecular basis of TTX biosynthesis is still unresolved. The first attempt to decipher the genes involved in TTX production was made by Liu et al. [3]. The authors suggested the TTX-producing ability of the bacteria *Aeromonas* sp. strain Ne-1 was associated with the copy number of plasmid pNe-1, containing 32 open reading frames encoding hypothetical proteins. However, the bacteria lost the plasmid after 18 h of culture. In the other work, the authors suggested an association between some natural product biosynthesis genes (polyketide synthase (PKS) and non-ribosomal peptide synthetase (NRPS)) and the TTX-producing ability of the microflora of toxic gastropods [4].

*Bacillus* sp. 1839 was isolated from the TTX-bearing nemertean *Cephalothrix* cf. *simula* in 2014 [5]. Confocal laser scanning microscopy with polyclonal antibodies against TTX allowed us to reveal TTX-positive labeling in the cells of the strain. Further detailed investigations with immunoelectron microscopy with anti-TTX antibodies revealed that toxin labeling was directly linked with the sporulation forms of the bacterium [6]. The life cycle and sporulation conditions studies [7,8] showed that TTX labeling was preserved through numerous passages for several years after the discovery of the strain. In 2019, the TTX producing ability of the strain was confirmed with high-performance liquid chromatography with tandem mass spectrometry [9]. TTX was revealed in the culture of the strain enriched with spores, confirming previous results.

A bacterial strain with TTX production in laboratory conditions is of great interest for the toxin biogenesis investigation. This study is the first to present the complete genome of a bacterium with in vitro TTX producing ability.

## 2. Results

### 2.1. General Genome Features of Bacillus sp. 1839

**Figure 1:**
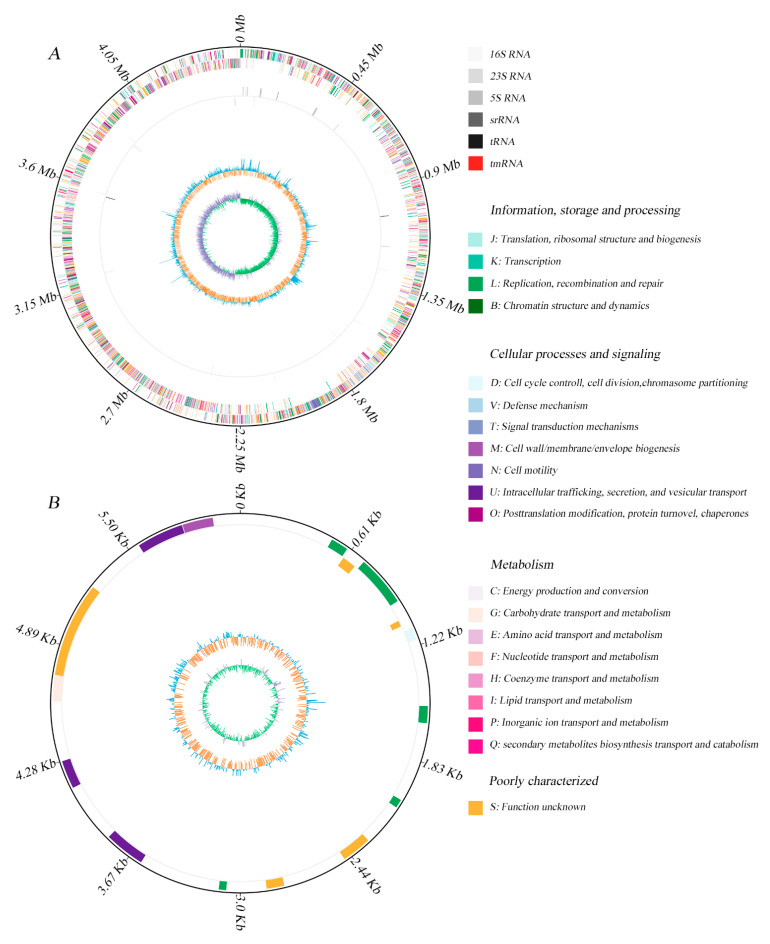
Graphic circular map of *Bacillus* sp. 1839 chromosome (**A**), and plasmid (**B**). From outer to inner rings, circle 1: the size of the complete genome; circle 2: the predicted protein-coding genes on the sense and antisense strands (colored according to Clusters of Orthologous Groups categories); circle 3: RNA genes; circle 4: G + C content, with >39% G + C in blue, with ≤39% G + C in orange; circle 5: G + C skew, with G% > C% in green, with G% < C% in violet.

The specific features of the *Bacillus* sp. 1839 genome are summarized in Table 1 and Figure 1. The genome of the strain consists of a single 4.5 Mb circular chromosome with 39% GC content (Figure 1A) and a 0.06 Mb plasmid with 34% GC content (Figure 1B). The *Bacillus* sp. 1839 genome is predicted to include 4527 total genes, of which 4369 (96.5%) are protein-coding genes, 119 (2.6%) are RNA-coding, and 39 (0.9%) are pseudogenes (Table 1). Among the predicted genes, 3508 (77.5%) are associated with general Clusters of Orthologous Groups (COG) function categories (Table 2), however, 22.8% of them are poorly characterized and are assigned to the S group with unknown functions. Among all COG groups, genes encoding transcription (K, 6.2%), amino acid transport and metabolism (E, 5.8%), inorganic ion transport and metabolism (P, 5.7%), and carbohydrate transport and metabolism (G, 4.9%) are the most abundant. Using the Kyoto Encyclopedia of Genes and Genomes (KEGG) pathway database, 2314 coding sequences (CDSs) of *Bacillus* sp. 1839 genome were assigned to 212 KEGG pathways (Supplementary Table S1). Among all the KEGG pathways, “Metabolic pathways” (547), “Biosynthesis of secondary metabolites” (254), “Microbial metabolism in diverse environments” (129), “Biosynthesis of amino acids” (104), “Biosynthesis of cofactors” (112), “Two-component system in signal transduction” (91), “ABC transporters” (90), and “Carbon metabolism” (81) accounted for the largest proportion. Comparative genomic analysis of *Bacillus* sp. 1839 using antiSMASH identified four gene clusters related to secondary metabolite production (Table 3).

Mobile genetic elements predicted in the genome of *Bacillus* sp. 1839 are summarized in Supplementary Table S2. Analysis of transposable elements revealed numerous insertion sequences (IS) distributed over the genome of the strain. The majority of IS belong to the IS1182 (76), followed by IS3 (10), IS21 (3), IS4 (1), IS110 (1), and IS1595 (1) families. Eight genomic islands (GEIs) are found in the genome with the IslandViewer4. The largest GEI is also assigned as an intact prophage by the PHASTER server. A total of two intact (score >90), one incomplete (score <70), and one questionable (score 70–90) prophage regions were predicted in the genome of the strain, indicating previous phage infection. Two clustered regularly interspaced short palindromic repeats (CRISPR) loci were also detected.

### 2.2. Phylogenetic Analysis and Genome Similarity Measures

**Figure 2:**
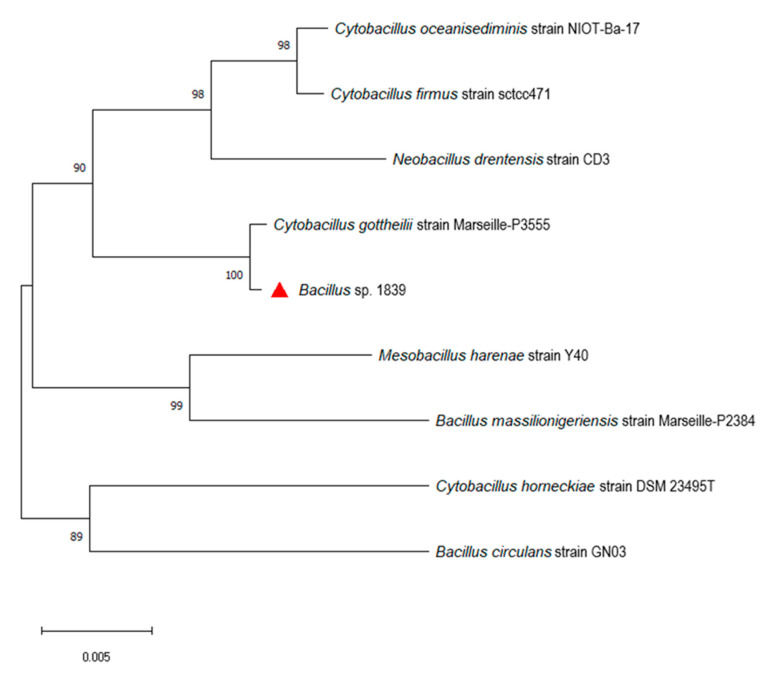
Neighbor-joining phylogenetic tree of *Bacillus* sp. 1839 and other closely related strains based on 16S rRNA gene sequences. Evolutionary distances are computed by the maximum likelihood method with 1000 bootstrap replications. Scale bar indicates 0.005 nucleotide substitutions per site. *Bacillus* sp. 1839 is indicated by the red triangle.

The phylogenetic tree based on 16S rRNA gene sequences revealed that *Bacillus* sp. 1839 is closest to *Cytobacillus gottheilii* (Figure 2). Up to now, only two complete genome sequences of this species have been deposited to the National Center for Biotechnology Information (NCBI) database (Table 4). We used these genomes for a more detailed analysis. As shown in Table 5, the closely related *Cytobacillus* strains resulted in a high average nucleotide identity (ANI) (>97%), exceeding the threshold value of 95% for distinguishing different species. Digital DNA–DNA hybridization (dDDH) values of *Bacillus* sp. 1839 against reference genomes were in the range of 79.6–85.9%, also exceeding the 70% DDH cutoff for species delineation (Table 6). This combination of analyses allowed us to classify *Bacillus* sp. 1839 as *Cytobacillus gottheilii*.

## 3. Discussion

In this study, we have described the genomic and phylogenetic features of the TTX-producing bacterium *Bacillus* sp. 1839. The phylogenetic analysis allowed to assign this strain to *Cytobacillus gottheilii*. The strains of this species were not earlier reported in TTX-production. Due to the complex structure of TTX, its biosynthetic pathway is not even predicted to date. It is assumed that the carbon backbone of TTX may originate through a polyketide, C5 branched sugar, or terpene [10]. The specific guanidinium moiety of the toxin can be obtained from a donor, such as an arginine, via amidinotransferase, similarly to amidino group transfer from l-arginine in saxitoxin biosynthesis [11], or NRPS and PKS systems [10]. The KEGG pathway database used to map the *Bacillus* sp. 1839 genome assigned 15 CDSs to “Arginine biosynthesis” and 17 CDSs to “Arginine and proline metabolism” indicating the potential ability of the bacterium to involve arginine in TTX production. Terpene and PKS gene clusters were also mined in the genome of the strain using antiSMASH. This study is the first to show the genome of a bacterial strain capable of TTX production in the laboratory—a good candidate for the unraveling of the molecular mechanisms of TTX synthesis. Genome sequencing is the first step allowing further experimental work aimed at gene cloning and expression, and reconstruction of the TTX synthetic and metabolic networks. The genome description and taxonomic classification opens the door to the comparative study of mutational patterns, ecological adaptations, and virulence determinants of the TTX producer and its closely related bacterial strains. Moreover, the genome obtained gives the possibility to reveal genes and traits not present in the other representatives of this species or, on the contrary, to find common features indicating their TTX-producing ability. Further investigations with the strain will be focused on the transcriptome studies on different stages of the life cycle of the bacterium.

## 4. Materials and Methods

### 4.1. DNA Extraction 

The strain *Bacillus* sp. 1839 (KF444411-KF444416) was previously isolated from the TTX-bearing nemertean *Cephalotrix* cf. *simula* (Ivata, 1952) [5]. For DNA analysis, a strain was obtained from the Collection of Marine Heterotrophic Bacteria, A.V. Zhirmunsky National Scientific Centre of Marine Biology, Far Eastern Branch of the Russian Academy of Sciences. The strain was aerobically cultivated in 2 mL of Youschimizu–Kimura liquid medium [5] at 23 °C overnight. Bacteria were centrifuged at 3000 g for 10 min. The bacterial pellet was suspended in 1 mL of lysis buffer containing 20 мM Tris-HCL (pH 8.0), 2 мM EDTA, 1.2% Triton X-100, and 20 mg/mL lysozyme, and incubated for 30 min at 37 °C. Genomic DNA of the strain was extracted using a GeneJET Genomic DNA Purification Kit #K0721 (Thermo Fisher Scientific, Waltham, MA, USA) according to the manufacturer’s instructions. For plasmid DNA extraction through Illumina sequencing, a GeneJET Plasmid Miniprep Kit # K0502 (Thermo Fisher Scientific, Waltham, MA, USA) was used. The DNA quality was evaluated using 1% agarose gel electrophoresis, an UV5Nano spectrophotometer (Mettler Toledo, Columbus, OH, USA), and a Qubit^®^ 2.0 Fluorometer (Thermo Fisher Scientific, Waltham, MA, USA). The optical density ratio at 260/280 nm and 260/230 nm was >1.8 and >2.0, respectively. DNA was stored at −20 °C until further processing.

### 4.2. Genome Sequencing

The complete genome of strain *Bacillus* sp. 1839 was sequenced by the Illumina HiSeq 2500 (Illumina Inc., San Diego, CA, USA) and MinIon (Oxford Nanopore Technologies, Oxford, UK) platforms. Sequencing on the Illumina HiSeq 2500 system was performed at Genoanalytica Company (Moscow, Russia). Genome DNA was fragmented by the Covaris M220 sonicator (Covaris, Woburn, MA, USA). Libraries were constructed using the NEBNext^®^ Ultra™ II DNA Library Prep Kit for Illumina^®^ (Illumina Inc., San Diego, CA, USA) for paired-end sequencing, with a target average insert size of 200 bp. For nanopore sequencing, genomic DNA was fragmented by passing through a small gauge needle. The fragmented genomic DNA was used to construct the library with the 1D Genomic DNA by ligation kit SQK-LSK108 following the instructions provided by the manufacturer. Sequencing was carried out on a MinION Mk1B sequencer (Oxford Nanopore Technologies) using an R9.4.1 Flow Cell. The read qualities were examined by FastQC.

### 4.3. Genome Assembly

Low quality reads from the Illumina HiSeq 2500 system were filtered out before de novo genome assembly by a St. Petersburg genome assembler (SPAdes) v. 3.7.1. [12] (kmer = 127), followed by genome finishing using the CONTIGuator tool v. 2.7 3. [13]. The filtered Illumina reads were used to improve the de novo assembly from the MinIon reads. The reads from the MinIon system were assembled by the Staden Package pipeline v. 2.0.0b11 (http://staden.sourceforge.net/ (accessed on 6 June 2021)). The final contigs were circularized by Unicycler v. 0.4.8 [14] and validated by BUSCO [15]. The final assembly was 98.3% complete with 0.4% of the sequence predicted to be missing, as estimated by BUSCO.

### 4.4. Genome Annotation

The complete genome sequence of *Bacillus* sp. 1839 was annotated using the NCBI Prokaryotic Genomes Automatic Annotation Pipeline (PGAAP). The gene functions were determined against the NCBI UniProt/Swiss-Prot and non-redundant (NR) protein databases, COG, Gene Ontology (GO), and KEGG databases with the E-value cutoff set to 10^−5^ and subsequent filtering for the best hit. tRNA and rRNA were identified by tRNAscan-SE v. 2.0 [16] and RNAmmer 1.2 [17], respectively. Circular representation of the genome including noncoding RNAs and gene function annotations was generated using the Circos software (version v.0.69-9) (http://www.circos.ca/ (accessed on 6 June 2021)). IS were predicted and classified with the ISFinder platform [18] against the ISfinder database v. 2.0 (http://www-is.biotoul.fr (accessed on 6 June 2021)). GEIs were detected with the IslandViewer4 online server using IslandPick, SIGI-HMM, and IslandPath-DIMOB prediction methods with default parameters [19]. CRISPR loci were detected using the CRISPRCasFinder online server [20]. Prophages in the genome were predicted with PHASTER online server [21,22]. The potential secondary metabolic gene clusters were predicted using antiSMASH v. 5.0 [23].

### 4.5. Phylogenetic Analysis and Genome Similarity Calculations

The 16S rRNA gene sequences of some strains closely related to *Bacillus* sp. 1839 were obtained by the BLASTN search against the NCBI database. The phylogenetic tree of Bacillus sp. 1839 and closely related species based on the 16S rRNA gene sequences was constructed using the neighbor-joining method [24] in MEGA X [25]. The evolutionary distances were computed using the maximum composite likelihood method [26] with 1000 bootstrap replications [27]. For genome similarity analysis, the genomes of two organisms closely related to the newly sequenced *Bacillus* sp. 1839 species were retrieved from the NCBI GenBank database (Table 4). The ANI values between the *Bacillus* sp. 1839 and closely related species *Cytobacillus* spp. were calculated using the BLASTALL algorithm (ANIb) and tetranucleotide frequency correlation coefficient (Tetra) with default parameters of the web server JSpecies v 1.2.1 [28]. Pairwise dDDH values were calculated using the Genome-to-Genome Distance Calculator (GGDC 2.1) [29].

## Figures and Tables

**Table 1:** The general genome features of *Bacillus* sp. 1839.

Feature	Value	
	Chromosome	Plasmid
Genome size (bp)	4,523,455	61,233
G + C content (mol %)	39	34
Genes number (total)	4458	69
Coding sequences total number	4339	69
Coding sequences with protein number	4300	69
Protein-coding genes number	4300	69
rRNAs number	11, 10, 10 (5S, 16S, 23S)	0
tRNAs number	83	0
ncRNAs number	5	0
Pseudogenes number	39	0
Insertion sequences number	92	0
Genomic islands number	9	0
Clustered regularly interspaced short palindromic repeats number	2	0
Prophage number	4	0

**Table 2:** Genes associated with general Clusters of Orthologous Groups (COG) functional categories of *Bacillus* sp. 1839.

Code	Value	% ^1^	Function Description
INFORMATION STORAGE AND PROCESSING			
B	1	0.02	Chromatin structure and dynamics
J	171	3.9	Translation, ribosomal structure, and biogenesis
K	269	6.2	Transcription
L	197	4.5	Replication, recombination, and repair
METABOLISM			
C	189	4.3	Energy production and conversion
E	255	5.8	Amino acid transport and metabolism
F	88	2	Nucleotide transport and metabolism
G	213	4.9	Carbohydrate transport and metabolism
H	100	2.3	Coenzyme transport and metabolism
I	88	2	Lipid transport and metabolism
P	248	5.7	Inorganic ion transport and metabolism
Q	29	0.7	Secondary metabolites biosynthesis, transport, and catabolism
CELLULAR PROCESSES AND SIGNALING			
D	36	0,8	Cell cycle control, Cell division, and chromosome partitioning
M	170	3.9	Cell wall/membrane/envelope biogenesis
N	43	1	Cell motility
O	113	2.6	Posttranslational modification, protein turnover, chaperones
T	191	4.4	Signal transduction mechanisms
U	38	0.9	Intracellular trafficking, secretion, and vesicular transport
V	75	1.7	Defense mechanisms
POORLY CHARACTERIZED			
S	994	22.8	Function unknown
-	848	19.5	Not in COGs

^1^ The total is based on the total number of protein coding genes in the genome.

**Table 3:** Putative gene clusters coding for secondary metabolites in *Bacillus* sp. 1839.

Type	From (bp)	To (bp)	Most Similar Known Cluster	% Similarity	Accession Number
Terpene	348,477	367,415	*Cytobacillus gottheilii* strain FJAT-2394	88	NZ_KV440945
Thiopeptide-Linear azol(in)e-containing peptides	447,791	476,894		82	
Terpene	973,108	1,001,908		89	
Type III polyketide synthase cluster	2,777,409	2,818,491		100	

**Table 4:** General genome features of the *Cytobacillus* spp. closely related to *Bacillus* sp. 1839.

Organism	NCBI Accession No.	Assembly Level	Size (bp)	GC (%)	Predicted Coding Sequences	No. of Genes	No. of Proteins	No. of RNAs
*Cytobacillus gottheilii* Marseille-P3555	NZ_FUVC00000000.1	Scaffold	4,719,939	39	4492	4621	4452	129
*Cytobacillus gottheilii* FJAT-2394	NZ_KV440945	Genome	4,584,535	39	4370	4475	4310	105

**Table 5:** Average nucleotide identity (ANI) and tetranucleotide frequency correlation coefficient (Tetra) analysis of *Bacillus* sp. 1839 and closely related *Cytobacillus* strains.

	*Bacillus* sp. 1839			*Cytobacillus gottheilii* Marseille-P3555			*Cytobacillus gottheilii* FJAT-2394		
	ANIb	Aligned	Tetra	ANIb	Aligned	Tetra	ANIb	Aligned	Tetra
*Bacillus* sp. 1839	*			97.55	85.95	0.99891	97.55	85.95	0.99892
*Cytobacillusgottheilii* Marseille-P3555	97.32	86.16	0.99891	*			100.00	99.49	1.0
*Cytobacillus gottheilii* FJAT-2394	97.34	86.18	0.99892	100.00	99.47	1.0	*		

* The asterisk indicates that the strain is compared to itself.

**Table 6:** Digital DNA–DNA hybridization (dDDH) analysis of *Bacillus* sp. 1839 with closely related *Cytobacillus* strains.

Query Genome	Reference Genome	Formula 1			Formula 2			Formula 3		
		DDH (%)	Model C.I. (%)	Distance	DDH (%)	Model C.I. (%)	Distance	DDH (%)	Model C.I. (%)	Distance
*Bacillus* sp. 1839	*Cytobacillus gottheilii* Marseille-P3555	83.7	79.8–86.9	0.1140	79.6	76.6–82.2	0.0239	85.9	82.8–88.5	0.1351
*Bacillus* sp. 1839	*Cytobacillus gottheilii* JAT-2394	83.7	79.9–86.9	0.1137	79.6	76.6–82.2	0.0239	85.9	82.8–88.6	0.1349

## Data Availability

The complete genome sequence of *Bacillus* sp. 1839 has been deposited in GenBank under the BioProject PRJNA707608 (accession number SRX10299645-SRX10299647). The annotated genome and plasmid are available in GenBank under accession numbers CP071709-CP071710.

## Supplementary Materials

The following are available online at https://www.mdpi.com/article/10.3390/toxins13060410/s1, Table S1: KEGG categories of *Bacillus* sp. 1839 genome, Table S2: Mobile genetic elements in *Bacillus* sp. 1839 genome.

## References

[1] Bane V., Lehane M., Dikshit M., O’Riordan A., Furey A. (2014). Tetrodotoxin: Chemistry, toxicity, source, distribution and detection. Toxins.

[2] Melnikova D.I., Khotimchenko Y.S., Magarlamov T.Y. (2018). Addressing the issue of tetrodotoxin targeting. Mar. Drugs.

[3] Liu J., Wei F., Lu Y., Ma T., Zhao J., Gong X., Bao B. (2015). Production level of tetrodotoxin in *Aeromonas* is associated with the copy number of a plasmid. Toxicon.

[4] Pratheepa V., Alex A., Silva M., Vasconcelos V. (2016). Bacterial diversity and tetrodotoxin analysis in the viscera of the gastropods from Portuguese coast. Toxicon.

[5] Beleneva I.A., Magarlamov T.Y., Kukhlevskii A.D. (2014). Characterization, identification, and screening for tetrodotoxin production by bacteria associated with the *Cephalotrix simula* (Ivata, 1952) proboscis worm. Mikrobiologiia.

[6] Magarlamov T.Y., Beleneva I.A., Chernyshev A.V., Kuhlevsky A.D. (2014). Tetrodotoxin-producing *Bacillus* sp. from the ribbon worm (Nemertea) *Cephalothrix simula* (Iwata, 1952). Toxicon.

[7] Shokur O.A., Magarlamov T.Y., Melnikova D.I., Gorobets E.A., Beleneva I.A. (2016). Life cycle of tetrodotoxin producing *Bacillus* sp. on solid and liquid medium: Light and electron microscopy studies. Russ. J. Mar. Biol..

[8] Magarlamov T.Y., Melnikova D.I., Shokur O.A., Gorobets E.A. (2017). Rapid production of tetrodotoxin-like compounds during sporulation in a marine isolate *Bacillus* sp. 1839. Microbiology.

[9] Melnikova D.I., Vlasenko A.E., Magarlamov T.Y. (2019). Stable tetrodotoxin production by *Bacillus* sp. strain 1839. Mar. Drugs.

[10] Chau R., Kalaitzis J.A., Neilan B.A. (2011). On the origins and biosynthesis of tetrodotoxin. Aquat. Toxicol..

[11] Kellmann R., Mihali T.K., Jeon Y.J., Pickford R., Pomati F., Neilan B.A. (2008). Biosynthetic intermediate analysis and functional homology reveal a saxitoxin gene cluster in cyanobacteria. Appl. Environ. Microbiol..

[12] Nurk S., Bankevich A., Antipov D., Gurevich A., Korobeynikov A., Lapidus A., Prjibelsky A., Pyshkin A., Sirotkin A., Sirotkin Y. (2013). Assembling genomes and mini-metagenomes from highly chimeric reads. J. Comput. Biol..

[13] Galardini M., Biondi E.G., Bazzicalupo M., Mengoni A. (2011). CONTIGuator: A bacterial genomes finishing tool for structural insights on draft genomes. Source Code Biol. Med..

[14] Wick R.R., Judd L.M., Gorrie C.L., Holt K.E. (2017). Unicycler: Resolving bacterial genome assemblies from short and long sequencing reads. PLoS Comput. Biol..

[15] Seppey M., Manni M., Zdobnov E.M. (2019). BUSCO: Assessing genome assembly and annotation completeness. Methods Mol. Biol..

[16] Chan P.P., Lowe T.M. (2016). tRNAscan-SE On-line: Integrating search and context for analysis of transfer RNA genes. Nucl. Acids Res..

[17] Lagesen K., Hallin P., Rødland E.A., Stærfeldt H.H., Rognes T., Ussery D.W. (2007). RNammer: Consistent annotation of ribosomal RNA genes. Nucl. Acids Res..

[18] Siguier P., Pérochon J., Lestrade L., Mahillon J., Chandler M. (2006). ISfinder: The reference centre for bacterial insertion sequences. Nucl. Acids Res..

[19] Bertelli C., Laird M.R., Williams K.P. (2017). IslandViewer 4: Expanded prediction of genomic islands for larger-scale datasets. Nucl. Acids Res..

[20] Couvin D., Bernheim A., Toffano-Nioche C., Touchon M., Michalik J., Néron B., Rocha E.P., Vergnaud G., Gautheret D., Pourcel C. (2018). CRISPRCasFinder, an update of CRISRFinder, includes a portable version, enhanced performance and integrates search for Cas proteins. Nucl. Acids Res..

[21] Arndt D., Grant J.R., Marcu A., Sajed T., Pon A., Liang Y., Wishart D.S. (2016). PHASTER: A better, faster version of the PHAST phage search tool. Nucl. Acids Res..

[22] Zhou Y., Liang Y., Lynch K.H., Dennis J.J., Wishart D.S. (2011). PHAST: A fast phage search tool. Nucl. Acids Res..

[23] Blin K., Shaw S., Steinke K., Villebro R., Ziemert N., Lee S.Y., Medema M.H., Weber T. (2019). antiSMASH 5.0: Updates to the secondary metabolite genome mining pipeline. Nucl. Acids Res..

[24] Saitou N., Nei M. (1987). The neighbor-joining method: A new method for reconstructing phylogenetic trees. Mol. Biol. Evol..

[25] Kumar S., Stecher G., Li M., Knyaz C., Tamura K. (2018). MEGA X: Molecular Evolutionary Genetics Analysis across computing platforms. Mol. Biol. Evol..

[26] Tamura K., Nei M., Kumar S. (2004). Prospects for inferring very large phylogenies by using the neighbor-joining method. Proc. Natl. Acad. Sci. USA.

[27] Felsenstein J. (1985). Confidence limits on phylogenies: An approach using the bootstrap. Evolution.

[28] Richter M., Rosselló-Móra R., Glöckner F.O., Peplies J. (2015). JSpeciesWS: A web server for prokaryotic species circumscription based on pairwise genome comparison. Bioinformatics.

[29] Meier-Kolthoff J.P., Auch A.F., Klenk H.-P., Göker M. (2013). Genome sequence-based species delimitation with confidence intervals and improved distance functions. BMC Bioinform..

