# Supplementary material for: The First Data on the Complete Genome of a Tetrodotoxin-Producing Bacterium
Source: Toxins (Basel). 2021 Jun 9;13(6):410. doi: 10.3390/toxins13060410 (PMC8228330; doi:10.3390/toxins13060410)
Supplement: Supplementary file 1 [file toxins-13-00410-s001.zip › Supplementary Table S1.pdf]

Supplementary Materials: The First Data on the Complete Genome of a Tetrodotoxin-Producing Bacterium

Daria I. Melnikova, Reindert Nijland and Timur Yu. Magarlamov

Table S1. KEGG categories of *Bacillus* sp. 1839.

No.	Value	Function Description
		METABOLISM
01100	547	Metabolic pathways
01110	254	Biosynthesis of secondary metabolites
01120	129	Microbial metabolism in diverse environments
01200	81	Carbon metabolism
01210	20	2-Oxocarboxylic acid metabolism
01212	17	Fatty acid metabolism
01230	104	Biosynthesis of amino acids
01240	112	Biosynthesis of cofactors
01220	5	Degradation of aromatic compounds
		**Carbohydrate metabolism**
00010	31	Glycolysis / Gluconeogenesis
00020	22	Citrate cycle (TCA cycle)
00030	23	Pentose phosphate pathway
00040	12	Pentose and glucuronate interconversions
00051	17	Fructose and mannose metabolism
00052	12	Galactose metabolism
00053	6	Ascorbate and aldarate metabolism
00500	25	Starch and sucrose metabolism
00520	26	Amino sugar and nucleotide sugar metabolism
00620	33	Pyruvate metabolism
00630	29	Glyoxylate and dicarboxylate metabolism
00640	27	Propanoate metabolism
00650	25	Butanoate metabolism
00660	7	C5-Branched dibasic acid metabolism
00562	4	Inositol phosphate metabolism
		**Energy metabolism**
00190	30	Oxidative phosphorylation
00195	8	Photosynthesis
00710	11	Carbon fixation in photosynthetic organisms
00720	24	Carbon fixation pathways in prokaryotes
00680	18	Methane metabolism
00910	10	Nitrogen metabolism
00920	11	Sulfur metabolism
		**Lipid metabolism**
00061	12	Fatty acid biosynthesis
00071	9	Fatty acid degradation
00072	4	Synthesis and degradation of ketone bodies
00100	1	Steroid biosynthesis
00561	9	Glycerolipid metabolism
00564	13	Glycerophospholipid metabolism
00600	1	Sphingolipid metabolism
00590	1	Arachidonic acid metabolism
00592	1	alpha-Linolenic acid metabolism
		**Nucleotide metabolism**

00230	44	Purine metabolism
00240	33	Pyrimidine metabolism
		**Amino acid metabolism**
00250	22	Alanine, aspartate and glutamate metabolism
00260	27	Glycine, serine and threonine metabolism
00270	35	Cysteine and methionine metabolism
00280	18	Valine, leucine and isoleucine degradation
00290	11	Valine, leucine and isoleucine biosynthesis
00300	15	Lysine biosynthesis
00310	9	Lysine degradation
00220	15	Arginine biosynthesis
00330	17	Arginine and proline metabolism
00340	15	Histidine metabolism
00350	5	Tyrosine metabolism
00360	7	Phenylalanine metabolism
00380	7	Tryptophan metabolism
00400	20	Phenylalanine, tyrosine and tryptophan biosynthesis
00410	6	beta-Alanine metabolism
00430	6	Taurine and hypotaurine metabolism
00450	8	Selenocompound metabolism
00460	5	Cyanoamino acid metabolism
00471	4	D-Glutamine and D-glutamate metabolism
00472	1	D-Arginine and D-ornithine metabolism
00473	3	D-Alanine metabolism
00480	8	Glutathione metabolism
		**Glycan biosynthesis and metabolism**
00531	2	Glycosaminoglycan degradation
00540	1	Lipopolysaccharide biosynthesis
00541	9	O-Antigen nucleotide sugar biosynthesis
00550	24	Peptidoglycan biosynthesis
00511	1	Other glycan degradation
00572	1	Arabinogalactan biosynthesis - Mycobacterium
		**Metabolism of cofactors and vitamins**
00730	15	Thiamine metabolism
00740	7	Riboflavin metabolism
00750	5	Vitamin B6 metabolism
00760	9	Nicotinate and nicotinamide metabolism
00770	16	Pantothenate and CoA biosynthesis
00780	5	Biotin metabolism
00785	4	Lipoic acid metabolism
00790	21	Folate biosynthesis
00670	11	One carbon pool by folate
00830	1	Retinol metabolism
00860	19	Porphyrin and chlorophyll metabolism
00130	10	Ubiquinone and other terpenoid-quinone biosynthesis
		**Metabolism of terpenoids and polyketides**
00900	13	Terpenoid backbone biosynthesis
00909	1	Sesquiterpenoid and triterpenoid biosynthesis
00906	5	Carotenoid biosynthesis
00981	1	Insect hormone biosynthesis
00908	1	Zeatin biosynthesis
00903	1	Limonene and pinene degradation
00281	2	Geraniol degradation
01051	1	Biosynthesis of ansamycins
00523	1	Polyketide sugar unit biosynthesis

01054	1	Nonribosomal peptide structures
01053	1	Biosynthesis of siderophore group nonribosomal peptides
	**Biosynthesis of other secondary metabolites**	
00940	1	Phenylpropanoid biosynthesis
00950	1	Isoquinoline alkaloid biosynthesis
00960	2	Tropane, piperidine and pyridine alkaloid biosynthesis
00966	2	Glucosinolate biosynthesis
00311	1	Penicillin and cephalosporin biosynthesis
00261	4	Monobactam biosynthesis
00521	4	Streptomycin biosynthesis
00524	1	Neomycin, kanamycin and gentamicin biosynthesis
00401	3	Novobiocin biosynthesis
00405	2	Phenazine biosynthesis
00333	3	Prodigiosin biosynthesis
00998	1	Biosynthesis of various secondary metabolites - part 2
	**Xenobiotics biodegradation and metabolism**	
00362	7	Benzoate degradation
00627	4	Aminobenzoate degradation
00625	3	Chloroalkane and chloroalkene degradation
00361	1	Chlorocyclohexane and chlorobenzene degradation
00622	2	Xylene degradation
00642	1	Ethylbenzene degradation
00643	2	Styrene degradation
00621	1	Dioxin degradation
00626	2	Naphthalene degradation
00980	1	Metabolism of xenobiotics by cytochrome P450
00982	1	Drug metabolism - cytochrome P450
00983	7	Drug metabolism - other enzymes
	GENETIC INFORMATION PROCESSING	
	**Transcription**	
03020	5	RNA polymerase
	**Translation**	
03010	55	Ribosome
00970	27	Aminoacyl-tRNA biosynthesis
03013	2	RNA transport
03008	1	Ribosome biogenesis in eukaryotes
	**Folding, sorting and degradation**	
03060	14	Protein export
04141	2	Protein processing in endoplasmic reticulum
04122	12	Sulfur relay system
03018	13	RNA degradation
	**Replication and repair**	
03030	15	DNA replication
03410	12	Base excision repair
03420	7	Nucleotide excision repair
03430	16	Mismatch repair
03440	19	Homologous recombination
03450	2	Non-homologous end-joining
	ENVIRONMENTAL INFORMATION PROCESSING	
	**Membrane transport**	
02010	90	ABC transporters
02060	22	Phosphotransferase system (PTS)
03070	12	Bacterial secretion system
	**Signal transduction**	
02020	91	Two-component system

04013	1	MAPK signaling pathway - fly
04016	3	MAPK signaling pathway - plant
04011	1	MAPK signaling pathway - yeast
04066	7	HIF-1 signaling pathway
04068	2	FoxO signaling pathway
04070	2	Phosphatidylinositol signaling system
04151	1	PI3K-Akt signaling pathway
04152	1	AMPK signaling pathway
	CELLULAR PROCESSES	
	**Transport and catabolism**	
04142	3	Lysosome
04146	7	Peroxisome
04138	1	Autophagy - yeast
	**Cell growth and death**	
04112	11	Cell cycle - Caulobacter
04216	2	Ferroptosis
04217	3	Necroptosis
	**Cellular community - prokaryotes**	
02024	35	Quorum sensing
05111	11	Biofilm formation - Vibrio cholerae
02025	7	Biofilm formation - Pseudomonas aeruginosa
02026	9	Biofilm formation - Escherichia coli
	**Cell motility**	
02030	15	Bacterial chemotaxis
02040	34	Flagellar assembly
	OTHERS	
04621	3	NOD-like receptor signaling pathway
04612	1	Antigen processing and presentation
04659	1	Th17 cell differentiation
04657	1	IL-17 signaling pathway
04910	2	Insulin signaling pathway
04922	7	Glucagon signaling pathway
04920	1	Adipocytokine signaling pathway
03320	2	PPAR signaling pathway
04915	1	Estrogen signaling pathway
04914	1	Progesterone-mediated oocyte maturation
04918	1	Thyroid hormone synthesis
04919	1	Thyroid hormone signaling pathway
04975	1	Fat digestion and absorption
04978	2	Mineral absorption
04724	1	Glutamatergic synapse
04727	1	GABAergic synapse
04211	2	Longevity regulating pathway
04212	5	Longevity regulating pathway - worm
04213	4	Longevity regulating pathway - multiple species
04714	3	Thermogenesis
04626	4	Plant-pathogen interaction
05200	2	Pathways in cancer
05206	1	MicroRNAs in cancer
05205	1	Proteoglycans in cancer
05204	1	Chemical carcinogenesis
05203	1	Viral carcinogenesis
05230	8	Central carbon metabolism in cancer
05211	1	Renal cell carcinoma
05215	1	Prostate cancer

05340	1	Primary immunodeficiency
05010	1	Alzheimer disease
05012	1	Parkinson disease
05014	3	Amyotrophic lateral sclerosis
05016	3	Huntington disease
05020	1	Prion disease
05022	3	Pathways of neurodegeneration - multiple diseases
05418	3	Fluid shear stress and atherosclerosis
04930	1	Type II diabetes mellitus
04940	1	Type I diabetes mellitus
04931	2	Insulin resistance
04934	1	Cushing syndrome
05120	1	Epithelial cell signaling in Helicobacter pylori infection
05130	1	Pathogenic Escherichia coli infection
05132	4	Salmonella infection
05131	1	Shigellosis
05134	4	Legionellosis
05150	1	Staphylococcus aureus infection
05152	3	Tuberculosis
05165	1	Human papillomavirus infection
05146	2	Amoebiasis
05143	1	African trypanosomiasis
01501	11	beta-Lactam resistance
01502	12	Vancomycin resistance
01503	3	Cationic antimicrobial peptide (CAMP) resistance
01524	1	Platinum drug resistance
01523	4	Antifolate resistance
